# Dr. Fred C. Beals

**Published:** 1914-10

**Authors:** 


Dr. Fred C. Beals, Buffalo, 1875, died at his home in Salamanca, July 25, aged 62.

